# The role of RAS oncogenes in controlling epithelial mechanics

**DOI:** 10.1016/j.tcb.2022.09.002

**Published:** 2023-01

**Authors:** Agata Nyga, Sushila Ganguli, Helen K. Matthews, Buzz Baum

**Affiliations:** 1 Medical Research Council Laboratory of Molecular Biology, Cambridge, CB2 0QH, UK; 2 Medical Research Council Laboratory for Molecular Cell Biology, University College London, London, WC1E 6BT, UK; 3 School of Biosciences, University of Sheffield, Western Bank, Sheffield, S10 2TN, UK; 4 Institute for the Physics of Living Systems, University College London, London, WC1E 6BT, UK

**Keywords:** RAS, oncogene, epithelia, mechanobiology, actin, myosin, mechanotransduction, YAP/TAZ signalling, tissue mechanics

## Abstract

Mutations in RAS are key oncogenic drivers and therapeutic targets. Oncogenic Ras proteins activate a network of downstream signalling pathways, including extracellular signal-regulated kinase (ERK) and phosphatidylinositol 3-kinase (PI3K), promoting cell proliferation and survival. However, there is increasing evidence that RAS oncogenes also alter the mechanical properties of both individual malignant cells and transformed tissues. Here we discuss the role of oncogenic RAS in controlling mechanical cell phenotypes and how these mechanical changes promote oncogenic transformation in single cells and tissues. RAS activation alters actin organisation and actomyosin contractility. These changes alter cell rheology and impact mechanosensing through changes in substrate adhesion and YAP/TAZ-dependent mechanotransduction. We then discuss how these changes play out in cell collectives and epithelial tissues by driving large-scale tissue deformations and the expansion of malignant cells. Uncovering how RAS oncogenes alter cell mechanics will lead to a better understanding of the morphogenetic processes that underlie tumour formation in RAS-mutant cancers.

## Highlights

Activation of RAS oncogenes and the downstream extracellular signal-regulated kinase/mitogen-activated protein kinase pathway alters actomyosin contractility, leading to changes in the mechanical properties of single cells and tissues.
Oncogenic RAS alters the ability of cells to sense the stiffness of their environment through changes to cell contractility and substrate adhesion.
Oncogenic RAS alters mechanotransduction via the YAP/TAZ signalling pathway.
Mechanical changes in RAS-activated cells can drive large-scale deformations in epithelial tissues, including buckling and folding.
The balance between elimination and preservation of RAS-transformed cells within a healthy epithelium is influenced by their differential mechanical phenotypes.

## RAS oncogenes drive tumorigenesis in many cancers

RAS was one of the earliest identified human oncogenes [1] and RAS family genes, KRAS (KRAS4A and KRAS4B), NRAS, or HRAS, the most commonly dysregulated proto-oncogenes in human cancer [2]. Ras proteins are small plasma membrane-associated GTPases that, under normal conditions, are activated by extracellular growth factors binding to surface transmembrane receptors [3]. Their activation induces multiple downstream signalling pathways, including the extracellular signal-regulated kinase 1/2 (ERK1/2) [4] and phosphatidylinositol 3-kinase (PI3K) cascades [5], to promote cell growth, cell cycle entry, and cell survival. Oncogenic RAS activation results in the hyperactivation of these pathways in the absence of a ligand or receptor activation to promote signal-independent cell proliferation and ultimately cancer. Thus, components of RAS-activated signalling have long been used as targets of anticancer therapies in the clinic [6]. Excitingly, after decades of being considered undruggable, oncogenic KRAS with an oncogenic G12C mutation has recently become a clinically important therapeutic target in its own right following the development of new mutation-specific inhibitors [7].

It has long been recognised that, unlike many other oncogenes, RAS signalling also alters the cytoskeleton [8] and cell adhesion [9]. These changes alter both the mechanical properties of cells and their ability to interact with and sense their mechanical environment. While many of these molecular changes have been studied in single cells, the effects on interconnected cells *in vivo* are profound. Multiple recent studies have demonstrated how RAS-dependent changes in cell mechanics result in large-scale deformations of epithelial tissues, including buckling and folding. These deformations are likely to be crucial in the loss of tissue architecture during tumorigenesis. In this review, we discuss how oncogenic RAS alters cell mechanics and mechanoresponses in single cells and how these changes translate to tissue-level disruption in epithelia and contribute to cancer progression.

## RAS alters cell contractility and mechanics

Cancer cells and tumours have material properties that are very different from those of healthy cells and tissues [10]. It is not clear when during tumorigenesis these internal changes arise and how much they are the product of evolution, because cancer cells adapt through mutation and selection to changes in their environment [11]. This includes their physical environment because tumours differ mechanically from healthy tissue due to extracellular matrix (ECM) stiffening [12], something known to promote invasion and metastasis [13]. It is also possible that oncogenic signalling itself changes cancer cell mechanics as a prerequisite to cancer cell survival and proliferation within the altered microenvironments they experience during tumour progression [14]. Because mutations that alter RAS signalling often occur early in cancer development [15], early oncogenic RAS mutations could set the stage for future cancer evolution.

**Figure 1:**
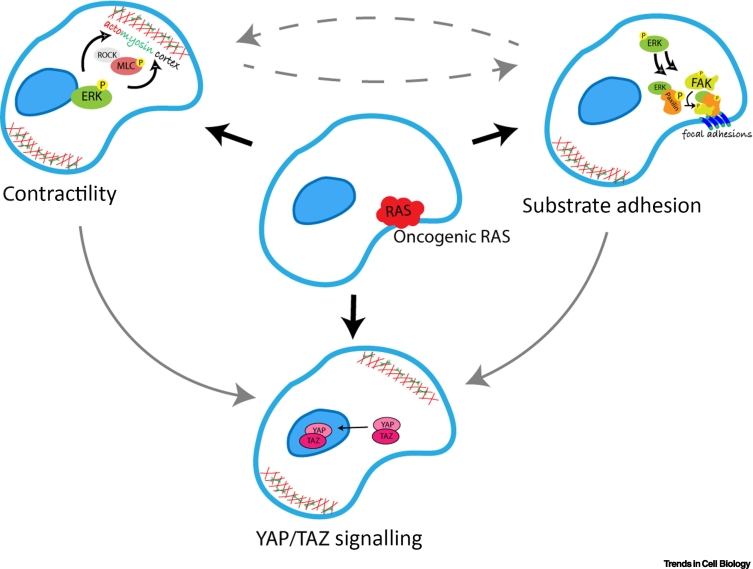
Oncogenic RAS-induced changes in cell cytoskeleton, mechanosensing, and signalling. Oncogenic RAS alters actomyosin contractility and focal adhesion assembly and disassembly. These processes in turn influence the activation and nuclear translocation of YAP/TAZ.

In support of this idea, oncogenic RAS has been shown to directly alter cell mechanics by altering cytoskeletal organisation and actomyosin contractility [16]. Oncogenic KRAS has been shown to increase contractility in mammary epithelial cells and their ability to exert forces on the substrate [17]. This was associated with an increase in actomyosin bundles, which are key in the generation and transmission of force. RAS-induced transformation has also been shown to require RhoA [8,18], which increases actomyosin contractility through the downstream effector ROCK and phosphorylation of myosin light chain [19]. Consistent with this, the contractile phenotypes seen in studies of RAS-transformed single cells and cell clusters are diminished by ROCK inhibition [17,20,21]. In addition, signalling pathways downstream of RAS, particularly the ERK/mitogen-activated protein kinase (MAPK) pathway, have been shown to modulate Rho GTPase activity and myosin contractility at multiple levels. Phosphoproteome analyses identified several Rho GTPase–activating proteins and Rho guanine nucleotide exchange factors (Rho GEFs) that are modulated by oncogenic RAS-ERK signalling [22]. In addition, a downstream substrate of growth factor–induced Ras-ERK signalling, the p90 ribosomal S6 kinase (RSK1 and RSK2), was shown to directly phosphorylate myosin phosphatase-targeting subunit 1 (MYPT1) to regulate cell migration in kidney cell lines [23]. A similar regulation of cell migration through RSK-MYPT1 and myosin activity was observed in the KRAS-mutant non–small cell lung adenocarcinoma cell line A549 and in the NRAS-mutant fibrosarcoma cell line HT1080 [23]. In addition, a study of glioblastoma cell lines (RAS wild type) reported a role for serum- and growth factor–induced ERK-RSK2 signalling in changing the cytoskeleton through the activation of RhoA through LARG, a Rho GEF, and actin binding proteins such as filamin A [24]. RAS-ERK was also shown to promote the nuclear translocation and activity of RSK2, which is required for transformation in this system [25]. These data demonstrating an impact of RAS on ROCK and RSK activity provide clear evidence of a direct path from oncogenic RAS-ERK signalling to reorganisation of the actomyosin cytoskeleton and cell contractility. Thus, oncogenic RAS and the deregulation of RAS-ERK signalling alter cell mechanics through multiple different mechanisms (Figure 1), some of which are likely to influence the ability of cells to undergo migration and invasion.

Oncogenic RAS has also been shown to alter the material properties of the cytoplasm. Measurements of cytoplasmic viscosity made using particle-tracking microrheology showed a decrease in particle movement within KRAS-transformed MCF10A cells [17]. Interestingly, despite this link between oncogenic RAS signalling and increased cytoplasmic viscosity and actomyosin contractility, individual cancer cells have frequently been found to be more compliant than nontransformed cells when mechanically probed [10,26,27], even in cases where overall tissue stiffness is increased [28]. Specifically, particle-tracking microrheology of lung adenocarcinoma cells showed that cancer cells within tissues soften, while the surrounding ECM stiffens [29]. In the case of RAS transformation, nontransformed breast epithelial cells (MCF10A) constitutively soften when forced to express oncogenic HRAS [14]. Similarly, a 24-h induction of oncogenic HRAS decreased the stiffness of loosely attached or suspended kidney epithelial cells (MDCK) in the absence of cell–cell adhesion [30]. However, in the same system, this increased the cortical stiffness of the monolayer as a whole. Thus, the effect of RAS on cell stiffness depends on whether RAS-transformed cells are isolated or present within a collective.

Because different oncogenic mutations have certain preferences for the downstream effectors, this could also modify the outcome of the RAS signalling network on the regulation of the cell mechanical response [5]. Cell compliance also depends on the cell adhesion to the substrate and other cells, which is altered by oncogenic mutations in RAS. As an example of this, increasing stiffness of a 3D collagen matrix in which cells are grown resulted in an increase in cytoplasmic elasticity and internal stiffness of single KRAS-mutant metastatic breast cancer cells (MDA-MB-231) measured by monitoring the thermal fluctuations of intracellular tracers with an optical trap [31]. Single cells detaching from MDA-MB-231 3D spheroids increased their cytoplasmic viscosity and therefore decreased their internal stiffness to facilitate migration [32]. This suggests a functional role of changes in cell compliance following RAS activation in facilitating processes such as cell migration and metastasis.

In addition, specific RAS-dependent changes in cell mechanics have been observed as cells round up and dissociate from the substrate before cell division. Mitotic rounding normally involves an actin-dependent increase in cortical tension [33] and cell stiffening [34,35]. However, the process can be accentuated by RAS transformation [14]. As a result, RAS-transformed cells entering mitosis round up better under conditions of physical confinement in a manner that depends on RAS-ERK signalling and the mitogen-activated protein kinase kinase (MEK).

## RAS alters cell–ECM interactions

Given the important role of the ECM in the regulation of cell mechanics, it is also important to consider the manifold ways in which RAS signalling alters mechanics through its impact on the ECM or cell–ECM attachment. RAS signalling alters the adhesion of cells to the ECM in part by impacting integrin-based substrate attachments at focal adhesion complexes [9,36] (Figure 1), altering cell behaviours to aid oncogenesis. As an example of this, in single mammary epithelial cells, oncogenic RAS-generated contractility increases the formation and maturation of focal adhesions and interferes with adhesion-driven mechanosensing through inhibition of focal adhesion kinase (FAK) [17]. In fibroblasts, binding of active ERK to focal adhesions by FAK and RACK1 allows their disassembly and facilitates cell migration [37]. At the molecular level, ERK associates with paxillin [38], and transient and sustained RAS-Raf-MEK–mediated ERK activation promotes paxillin phosphorylation [39], which is necessary for its tyrosine phosphorylation and association with FAK at focal adhesions [40]. ERK regulation of the paxillin–FAK complex at focal adhesions also increases the association of FAK with p85, a subunit of PI3K, leading to the activation of downstream kinase Akt. This activation of PI3K results in further activation of Rac GTPases [38], which play a major role in control of the actin cytoskeleton. Rac activation, together with Cdc42 and myosin II, forms a key response to mechanical stress in KRAS-mutated pancreatic cancer cells, promoting cytoskeletal remodelling, contractility, and migration [41]. In the absence of attachment to a substrate anchorage-independent growth of RAS-transformed cells also requires paxillin-regulated FAK phosphorylation [42]. At the same time, active RAS can act as a negative focal adhesion regulator by mediating the dephosphorylation of both paxillin and FAK at the Y397 site, a process that is regulated not by Raf or PI3K but by direct activation of Cdc42 and PAK1 [43]. Thus, the tightly regulated processes of focal adhesion assembly and disassembly are key to many RAS-induced oncogenic cell behaviours, including cell transformation, migration, and invasion.

Because adhesion affects the cytoskeletal remodelling in adherent cells and vice versa, it is hard to dissect the direct cause of RAS-induced changes to the mechanical responses of cells in complex environments. One study looking at the impact of changes in substrate stiffness to the behaviour of KRAS-transformed cells [17] showed that cells spread on soft substrates (150 Pa) in an ERK-dependent manner, but not on stiffer substrates (5.7 kPa), closer to those found in fibrotic tumours [44]. In this case, the inhibition of myosin by the treatment of cells with blebbistatin did not impact cancer cell spreading, implying a role for myosin-independent regulators of cell spreading, such as cell–substrate adhesion, in this change in cell spreading behaviour.

Taken together, these data show that oncogenic RAS has an impact on the material properties of the cytoplasm, the cell cortex, and the extracellular material in a tissue.

## Oncogenic mechanosensing and mechanotransduction

RAS has also been shown to affect the ability of cells to sense their mechanical environment. Mechanosensation, or mechanosensing, depends in part on integrin-based substrate attachments [36] and is important for allowing cells to modify their own stiffness through the reorganisation of the actin cytoskeleton in a manner that is suited to the mechanical environment in which the cells find themselves. It also requires active contractility. As an example of the impact of oncogenic mutations on mechanosensation, one study found that KRAS-transformed cells were more sensitive to changes in substrate stiffness than their nontransformed counterparts [17]. This shows that oncogenic signalling does not always disrupt the sensitivity of cancer cells to their environment. RAS-ERK signalling also crosstalks with the YAP/TAZ pathway. YAP/TAZ transcriptional regulators play critical roles in mechanotransduction [45], and their role in cancer development and progression has been widely studied and previously reviewed [46,47]. YAP/TAZ are transcriptional coactivators of the Hippo pathway that shuttle between the cell cytoplasm and nucleus in response to mechanical cues, such as the activation of Rho and cortical tension [45]. Translocation to the nucleus allows their binding to transcription factors and control of tissue homeostasis through the regulation of cell proliferation, apoptosis, and stem cell renewal. The induction of YAP/TAZ nuclear shuttling by oncogenic RAS suggests a direct link between the RAS and Hippo signalling pathways and a possible synergistic role in oncogenic transformation. This is of particular importance because experiments in mice have shown that relapsed KRAS pancreatic tumours have activated YAP1/TEAD2 transcriptional programs that are required for tumour growth [48]. In addition, the overexpression of YAP1 has been shown to clinically correlate with metastasis and poor prognoses in patients with pancreatic ductal adenocarcinoma (PDAC) [49].

YAP is also translocated to the nuclei of cells in response to their exposure to a stiffer microenvironment and following a direct application of force on the cell nucleus. In both cases, this results in nuclear flattening, which increased the passive transport through nuclear pores, perhaps as the result of mechanically induced nuclear pore dilation [50]. Whether cell spreading following RAS activation results in the flattening of the nucleus and associated increase in YAP translocation is still not clear. However, in one study, it was shown that once YAP has been activated on substrates stiffer than 1 kPa, ERK inhibition no longer impacts cell spreading. This implies that the two pathways can act in parallel [17]. In the context of cancer, the expression and nuclear translocation of YAP were also shown to be altered by constitutive activation of PI3K and the downstream effector phosphoinositide-dependant kinase (PDK1) [51,52], post-transcription modifications through the RAS-MAPK pathway [53], and direct phosphorylation of TEAD3 through increased oncogenic RAS-ERK signalling [22]. Particularly, there is growing evidence supporting the mechanotransduction role of PI3K-PDK1 in regulating YAP/TAZ signalling in development [54] and oncogenesis [55].

Although these data show that oncogenic RAS modulates cell–substrate interactions and mechanosensing, most of these studies have been carried out in single or sparsely plated cells. How do changes observed at the single-cell level contribute to RAS oncogenesis in the context of tissues *in vivo*?

## Oncogenic RAS (mis)shapes tissues

**Figure 2:**
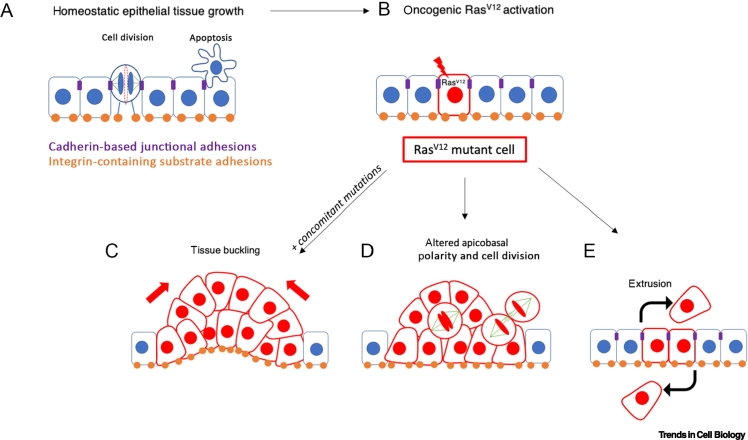
RAS-driven alterations in cell mechanical properties drive tissue transformation. (A) Normal epithelial tissue morphogenesis and homeostasis result from a balance between orientated cell division and cell death. (B) Oncogenic mutations in RAS can drive the misshaping of oncogenic tissue, which can acquire other common mutations. (C) Oncogenic RAS drives cell proliferation and hyperplastic growth. Subsequent changes in cellular and substrate stiffness can lead to tissue compaction and buckling. (D) RAS-driven changes in cell contractility and adhesion can also impact tissue polarity and division mechanics. (E) RAS mutations initiate cell competition within tissue, which is regulated by mechanical balance between the neighbours.

The oncogenic transformation of an epithelial tissue occurs within spatial constraints imposed by cell neighbours and the ECM [56]. It is therefore important to determine how oncogenic RAS-induced changes to actomyosin contractility, cell mechanics, substrate adhesion, and mechanotransduction in individual cells play out at the level of the collective or tissue. In this section, we discuss how oncogenic RAS impacts the cellular and mechanical changes at the tissue scale (Figure 2).

Activation of RAS within cell collectives can induce large-scale morphological tissue movements. Induction of oncogenic HRAS in confined human breast epithelial monolayers resulted in the cell monolayer compacting towards a 3D aggregate, a process caused by a mechanical instability at the tissue level and changes in the distribution of cellular tension [20]. This correlated with an increase in cell contractility and myosin phosphorylation. Activation of oncogenic KRAS also disrupted the morphology of 3D colonic epithelial cysts [57]. Although monolayer compaction was prevented by inhibition of myosin or ROCK activity, cyst polarity was partially restored upon inhibition of the ErbB3 receptor through the control of growth factor heregulin (HRG) signalling. Interestingly, HRG causes sustained activation of Rac, Cdc42, and RhoA [58], indicating that changes in the mechanical properties of cells are key in complex 3D malignant transformations (Figure 2C). In addition, the levels of oncogenic KRAS determine the growth dynamics and aggressiveness of cancer cells with other mutations, such as *Lkb1* (the third most frequently mutated tumour suppressor in human lung adenocarcinoma) [59]. Loss of *Lkb1* alone within the wild-type epithelium results in a high level of apoptosis, loss of *Lkb1* and low KRAS expression promote benign overgrowth, and loss of *Lkb1* and high levels of oncogenic KRAS promote neoplastic transformation [59]. KRAS mutation also drives basal invasion of transformed cells in zebrafish, and acquisition of p53 mutations promotes their survival and migration, behaviours that are inhibited in the presence of Rho kinase inhibition [60].

The role of actomyosin contractility in the shaping of transformed tissue has also been demonstrated in mouse models. The mosaic activation of oncogenic KRAS alongside deletion of the tumour suppressor p53 or Fbw7 in pancreatic ducts resulted in ductal size-dependent formation of two types of distinct oncogenic lesions that formed either basal or apical evaginations [61]. Importantly, similar findings were observed in pancreatic biopsies of patients with PDAC [61]. In a model, hyperproliferation of cells alone was not able to recapitulate the form of these oncogenic lesions unless polarised actomyosin activity was also included. These phenotypes were altered by inhibiting pMLC2 expression [61]. Importantly, in PDAC, it was the basal-like phenotype that correlated with aggressive and invasive oncogenic lesions [62]. HRAS has also been shown to drive the formation of abnormal basal folds with an invaginated apical surface during early morphogenesis of squamous cell carcinoma [63]. These were associated with increased tension at the basement membrane through suprabasal stiffening [63]. These types of responses seem to be conserved across animal evolution, because the ectopic expression of RAS in *Drosophila* wing imaginal discs induces hyperplasia, overgrowth of the tissue, and the formation of ectopic folds [64]. These observations show that, although the biophysical alterations can differ between stratified and simple epithelia, transformed cells are key drivers of epithelial deformation, leading to acquired tumour morphology in each case. Such functions in the context of epithelia may explain the role played by the Rho-ROCK pathway in RAS-driven tumorigenesis and metastasis, making it a potential therapeutic target in RAS mutant cancers [65,66]. In both human and mouse KRAS-driven tumours, an increase in tumour progression correlates with elevated levels of ROCK1/ROCK2 kinases [67,68]. Targeting the Rho-ROCK pathway reduces tumour growth and blocks invasion of the healthy pancreatic tissue by transformed cells [69].

The response to RAS activation within tissues also depends on the size of the group of cells in which RAS is activated. When initial oncogenic mutations occur in a limited number of cells within a nontransformed background, mutant cells can be eliminated due to cell competition [70] in processes that are dependent on ROCK [71] and YAP/TAZ signalling [72]. Moreover, tumour cell survival has been shown to depend on the relative activity of YAP and TAZ in tumour cells and the surrounding tissue [72]. Thus, whereas hyperactivation of YAP/TAZ signalling is associated with cancer development, the accumulation of YAP/TAZ in the peritumoral hepatocytes in a cholangiocarcinoma mouse model was found to reduce tumour burden by triggering cell death in cancer cells. This tumour-suppressive mechanism was dependent on the differential activation of YAP/TAZ signalling between tumour and normal hepatocytes and was sufficient to also eliminate NRAS-mutant melanoma metastases from the liver. In a lung cancer model, YAP activity is essential for KRAS/p53-driven tumorigenesis, and heterogeneous expression of YAP correlates with cancer cell proliferation [73]. Cell competition can also lead to a number of outcomes that involve changes in cell mechanics. Differences in the mechanical sensitivities between cancer and noncancer cells can lead to cell death [71], and the hyperactivation of RAS can result in the extrusion of transformed cells from an epithelium [74] (Figure 2E). It is clear, however, that this is not a fail-safe mechanism, because epithelia are able to accumulate cells with somatic mutations [75], and retention of activated RAS cells throughout an epithelial monolayer can lead to whole-tissue morphogenesis and hyperplasia [20]. This may be due to the role of mechanics in the extrusion process, because experimental work has shown that the extrusion of transformed RAS cells from monolayers is reduced by the application of external strain, which instead promotes basal invasion, partially through activation of the Rho-ROCK pathway and FAK [76]. Extrusion is also weakened by matrix stiffening. On substrates stiffer than 11 kPa, similar to fibrotic tissue, the number of HRAS-transformed MDCK cells undergoing extrusion drastically decreases. Stiffer matrices also prevent filamin, an actin filament crosslinking protein, from moving to the interface between wild-type and transformed cells [77]. This has consequences for cell extrusion, because filamin is required in wild-type cells for successful extrusion of neighbouring transformed cells [78]. Accumulation of filamin at the perinuclear regions results in inhibition of force generation required by wild-type cells to extrude HRAS-transformed cells [77]. This may in part explain why HRAS-transformed cells remain within epithelia on stiff substrates [77], and it may also help to explain why tissue fibrosis is a risk factor in the development and progression of many RAS-driven cancers, including KRAS mutant PDAC [79].

The mechanical impact of cancer cell growth can also be felt more widely in a tissue. In the *Xenopus laevis* embryonic epithelium, clusters of KRAS-expressing cells form tumour-like structures characterised by high contractility and tension [21]. This tension originates not at the boundary of KRAS and wild-type cells, but from mutant KRAS cell clusters whose increased cortical contractility is sufficient to impact the orientation of dividing cells in the surrounding wild-type tissue [21] (Figure 2D). In a *Drosophila* model, activation of oncogenic RAS drives the progressive downregulation of ERK signalling in wild-type neighbours through a reduction in cell tension. This leads to the increased compaction, apoptosis, and elimination of wild-type cells, facilitating the expansion of transformed clones [80]. In models of human cancer, the unconstrained growth of transformed cells can lead to the killing of nonadjacent wild-type cells through the induction of mechanical compression [81].

These studies demonstrate how changes in contractility and mechanosensing downstream of RAS oncogenes can induce tissue-level disruptions. In the future, it will be important to investigate how these mechanical changes influence tissue growth and structure in a more complex human tissue or tumour microenvironment.

## Concluding remarks

RAS oncogenes are key driver mutations in the tumorigenesis of many cancers. Integrating knowledge of downstream signalling pathways activated by oncogenic RAS and the mechanical alterations that occur at both the cell and tissue levels will play a pivotal role in determining the contribution of mechanoresponses in cancer initiation and progression. Oncogenic RAS activation in individual cells *in vitro* impacts actomyosin organisation and cell compliance, but it is not yet clear how far these changes are reflected in RAS-mutant cancer cells *in vivo* and how they influence tumour growth and metastasis. Similarly, it is not known how RAS-induced changes to mechanotransduction pathways, such as YAP/TAZ signalling, would contribute to rigidity sensing within a complex and heterogeneous tumour microenvironment. Addressing these questions will require the application of new imaging approaches (e.g., Brillouin microscopy [82]) to accurately measure tissue and tumour rheology *in vivo*. Over tissue scales, mechanical alterations following RAS activation contribute to a wide variety of morphogenetic perturbations from the extrusion of oncogenic cells to hyperplasia, altered cell division mechanics, and large-scale tissue deformation. The tissue mechanoresponse to RAS activation is highly context and tumour-type specific. In the future, it will be important to determine what controls whether malignant cells expand or become extruded (e.g., RAS expression levels, critical mass of mutant cells, properties of the microenvironment) and whether there is a way to tip the balance in favour of elimination. Ultimately, a better understanding of the contribution of RAS to the interplay between oncogenic cells and their surrounding microenvironment in the context of disease-specific tumour models could potentially influence the development of new therapeutic strategies in preventing cancer progression. Combining RAS-targeted treatments (either with direct RAS isoform inhibitors or by targeting downstream signalling pathways, e.g., ERK) with mechanotherapeutics (e.g., targeting ROCK pathway, mechanosensing machinery) could provide the potential for effective treatments of RAS-driven tumours. As such, early promising synergistic effects of targeting RAS and FAK have been observed in several cell lines and patient-derived xenograft models [83], with several clinical trials taking place currently [84] (see Outstanding questions).

Outstanding questions How do RAS-driven changes in the mechanical properties of individual cells measured *in vitro* manifest *in vivo* within a tumour microenvironment? How do they influence tumour growth and aggressiveness?

What determines the mechanoresponse of tissues to RAS-activated cells and whether oncogenic cells expand or become eliminated by healthy tissue structure?

Can the RAS mechanoresponse be targeting therapeutically? For example, will pharmacological targeting of RAS or downstream pathways together with the mechanosensing pathways provide effective treatments for RAS-driven tumours?

How do the precise wiring of RAS signalling and downstream mechanical signalling differ across human tumours?

Alt-text: Outstanding questions
